# Childhood maltreatment and adulthood domestic and sexual violence victimisation among people with severe mental illness

**DOI:** 10.1007/s00127-016-1244-1

**Published:** 2016-05-28

**Authors:** Fraser Anderson, Louise Howard, Kimberlie Dean, Paul Moran, Hind Khalifeh

**Affiliations:** Health Services and Population Research, Section of Women’s Mental Health, King’s College London, PO31 David Goldberg Centre, IOPPN, De Crespigny Park, London, SE5 8AF UK; School of Psychiatry, University of New South Wales, Sydney, Australia; Justice and Forensic Mental Health Network, Matraville, Australia; Centre for Academic Mental Health, School of Social and Community Medicine, University of Bristol, Bristol, UK

**Keywords:** Mental health, Domestic violence, Sex offenses, Crime victims, Trauma

## Abstract

**Purpose:**

To investigate the association between childhood maltreatment and adulthood domestic and sexual violence victimisation among people with severe mental illness (SMI), and to explore this association in terms of gender differences and potential mediators.

**Method:**

A cross-sectional survey of 318 people living in the community who were receiving care from Community Mental Health Teams. Associations were assessed using logistic regression of multiply imputed data.

**Results:**

63 % (95 % CI 55–71 %) of men and 71 % (95 % CI 63–79 %) of women reported childhood maltreatment, 46 % (95 % CI 37–54 %) of men and 67 % (95 % CI 59–76 %) of women reported adulthood domestic violence victimisation, and 22 % (95 % CI 15–28 %)of men and 62 % (95 % CI 53–70 %)of women reported adulthood sexual violence victimisation. Men and women with SMI who reported experiences of childhood maltreatment were two to five times more likely to report domestic and sexual violence victimisation in adulthood after adjusting for confounders. The associations held for each of emotional, physical and sexual childhood abuse.

**Conclusion:**

People with severe mental illness have high prevalence of experiences of childhood maltreatment and adulthood domestic and sexual violence victimisation. Childhood maltreatment appears to be an independent risk factor for adulthood victimisation among men and women with SMI.

## Introduction

People with severe mental illness (SMI) are at increased risk for all forms of violent victimisation [1]. Domestic and sexual violence victimisation is common among people with SMI, and victims show higher levels of psychosocial morbidity following violence than in the general population [2]. Although, there is an established association between childhood maltreatment and adulthood violent victimisation in the general population [3, 4], there is little evidence for the SMI population. People with SMI are a particularly vulnerable population, suffering from a range of mental and physical morbidity, social disadvantage, and elevated risk of premature mortality [5, 6]. It is therefore, particularly important to advance our understanding of early risk factors for later difficulties in this population as this might help pave the way for preventative interventions.

Childhood maltreatment refers to both childhood abuse (emotional, physical and sexual) and childhood neglect (emotional and physical). In the general population there is strong evidence for the association between childhood abuse and adult abuse and trauma, even after adjustment for confounding variables [3]. A prospective cohort of abused and neglected children with matched controls found that the increase in risk of adult victimisation associated with childhood abuse and neglect was specifically an increase in risk of interpersonal violence such as physical and sexual assault/abuse [4]. All types of childhood abuse and neglect increased the risk of interpersonal violence in adulthood with no evidence for specific associations between subtypes of childhood maltreatment and specific forms of victimisation in adulthood [4]. Although, the same pattern of increased risk was observed for men and women with experiences of childhood maltreatment, the effect on increased risk for the event “coerced into unwanted sex” was significantly stronger for men than for women [4]. In both the general population and among people with SMI, women are at greater risk for domestic and sexual violence [7–9] and thus, risk factors and potential mediators for intimate violence must be explored by gender.

As the cause of domestic and sexual violence is always ultimately the behaviour of the perpetrator, it can be difficult to clarify the mechanisms by which a person’s negative childhood experiences could increase their vulnerability to later violence. Grauerholz uses an ecological framework, proposing that personal, interpersonal and sociocultural factors associated with childhood abuse may increase the risk of exposure to potential perpetrators, or increase the likelihood that potential perpetrators will act aggressively [10]. Factors associated with childhood abuse in the general population such as lack of resources, social isolation, drug and alcohol abuse, psychiatric symptoms and stigmatization [11–13] may all increase the risk of a perpetrator acting aggressively, due to the perception of the victim as an easy target and feeling more justified in behaving aggressively, as well as decreasing the ability of the victim to respond assertively [10]. Many of the factors considered to be the potential mediators of the relationship between childhood abuse and adult victimisation are very prevalent in populations with SMI, regardless of abuse history.

The prevalence of childhood maltreatment among people with SMI is extremely high [14, 15]. Experiences of childhood maltreatment are associated with more severe psychiatric symptoms and more complex clinical manifestations among people with SMI [16, 17]. People with SMI also have a much higher prevalence of both past-year and lifetime experiences of domestic and sexual violence compared to general population samples [8, 18–20]. Sexual and domestic violence among people with SMI is associated with substance abuse, homelessness, psychiatric illness severity and history of childhood abuse [8, 21].

Despite the high prevalence of victimisation across the lifetime, and the association of victimisation with psychopathology, there have been very few studies which have looked at the association between childhood maltreatment and domestic and sexual violence in adulthood among populations with SMI. The studies that have been conducted to date have often excluded men with SMI, and have not adequately adjusted for confounding factors. In addition, previous studies have not investigated associations between adult victimisation status and the occurrence of different forms of childhood maltreatment and abuse [21–24].

### Aims of the study

We aimed to explore the association between childhood maltreatment and adulthood sexual and domestic violence victimisation among people with SMI, investigating gender differences, potential mediating factors, and the risk associated with different forms of childhood maltreatment. Our primary hypothesis was that the experiences of moderate to severe childhood maltreatment would increase the odds of adulthood domestic and sexual violence victimisation among both men and women with SMI.

## Method

### Study design and setting

The study design was a cross-sectional survey. The sample was drawn using simple random sampling from 19 community mental health teams (CMHTs) based in two NHS mental health Trusts, covering six London boroughs with a diverse population of 1.5 million people. Interviews were conducted from September 2011 to March 2013 by trained research workers. The interviews lasted around an hour and participants were paid £20 for their time. The survey instrument comprised a modified version of the Office for National Statistics (ONS) Crime Survey for England and Wales (CSEW) questionnaire, comprising a main face-to-face interview and a self-completion questionnaire.

### Study population

Severe Mental Illness (SMI) was defined in terms of chronicity and need for intensive care from secondary mental healthcare services, in accordance with the UK Department of Health definitions [25]. Patients were eligible for the study if they were aged 18–65, receiving secondary mental health care from one of the included CMHTs for 1 year or more, and living in the community. Patients were excluded if they had poor English language proficiency or lacked capacity to consent.

### Study variables

#### Exposure

The primary exposure was the experience of childhood maltreatment, assessed using the Childhood Trauma Questionnaire-Short Form (CTG-SF) [26]. The CTQ-SF is a well-validated, 28-item, self-report questionnaire. Individuals respond to a series of statements about childhood events, which are endorsed on a Likert scale according to their frequency. The CTQ-SF has three subscales for emotional, physical and sexual abuse; and two for emotional and physical neglect. The CTQ subscales were based on the following definitions of abuse and neglect:

- *Sexual abuse* Sexual contact or conduct between a child younger than 18 years of age and an adult or older person.
- *Physical abuse* Bodily assaults on a child by an adult or older person that posed a risk or resulted in injury.
- *Emotional abuse* Verbal assaults on a child’s sense of worth or well-being or any humiliating or demeaning behaviour directed toward a child by an adult or older person.
- *Physical neglect* The failure of caretakers to provide for a child’s basic physical needs, including food, shelter, clothing, safety and health care.
- *Emotional neglect* The failure of caretakers to meet children’s basic emotional and psychological needs, including love, belonging, nurturance, and support.

The CTQ-SF has cut scores set for each type of maltreatment at four levels: None (or minimal), Low (to Moderate), Moderate (to Severe) and Severe (to Extreme). For the purpose of this study a binary outcome for each type of maltreatment was created using the Moderate to Severe cut off. This cut score achieves a specificity of at least 95 % of non-maltreatment cases correctly classified, and has sensitivities ranging from 49 to 72 % [26].

#### Outcome

The two main outcomes in this analysis were experiences of adulthood domestic violence victimisation (ADVV) and adulthood sexual violence victimisation (ASVV). Domestic violence refers to all non-sexual violence perpetrated by an intimate partner or family member, and sexual violence refers to violence of a sexual nature regardless of the perpetrator (ONS 2013).

ASVV was assessed in the self-completion module of the survey, with the following four questions, the same questions used in the CSEW:

- *Indecent exposure* “Since you were 16, has ANYONE ever indecently exposed themselves to you (i.e. flashing) in a way that caused you fear, alarm or distress?”
- *Sexual touching* “Since you were 16, has anyone ever touched you in a sexual way (e.g. touching, grabbing, kissing or fondling) when you did not want it?”
- *Sexual intercourse* “Since you were 16, has anyone ever forced you to have sexual intercourse, when you were not capable of consent or when you made it clear you did not want to? By sexual intercourse we mean vaginal, anal or oral penetration.”
- *Attempted sexual intercourse* “Apart from anything else you have already mentioned, since you were age 16 has anyone ever ATTEMPTED to force you to have sexual intercourse when you were not capable of consent or when you made it clear you did not want to?”

For each question, if the patient answered “Yes”, they were asked to specify the perpetrator: a partner, a family member (other than a partner), someone else I knew (other than a partner of family member), a stranger. As well as a “No” option, there was a “Do not know/cannot remember/do not wish to answer” option. For each question the patient was also asked if anyone had done this to them in the last 12 months, and were again asked to specify the perpetrator. We used a binary variable of ASVV (yes/no) for all analyses, so anyone who endorsed any one of the four sexual violence questions was coded as having experienced ASVV.

ADVV was assessed with the following questions for partner or ex-partner violence and the questions were repeated for family violence.

- Since you were 16 has a partner or ex-partner (/member of your family) EVER done any of the things listed below?
  - Prevented you from having your fair share of the household money.
  - Stopped you from seeing friends and relatives.
  - Repeatedly belittled you to the extent that you felt worthless.
- Since you were 16 has a partner or ex-partner (/member of your family) EVER threatened you in any way?
- Since you were 16 has a partner or ex-partner (/member of your family) EVER used a force on you.
- Have you EVER been injured (even if only slightly) as a result of the force used on you by a partner (/member of your family)?

Again, if responded positively, the participant was asked to specify whether this occurred in the past 12 months, and a binary variable of experienced any ADVV was used for all analyses.

#### Covariates

We included co-variates that were hypothesised to be a priori confounders or mediators on the basis of previously published studies [19, 21]. The confounders adjusted for age were, ethnicity and the social class of the household respondent. The variables adjusted for the final model including potential mediators were social variables (employment status, social support, living alone, history of perpetration of violence) and clinical variables (illness severity using primary ICD-10 diagnosis and history of admission under the mental health act (MHA) as markers, and alcohol and substance misuse) thought to potentially lie on the causal pathway between childhood maltreatment and adulthood victimisation.

##### Demographic

Basic demographic information on age, gender, ethnicity, education status, employment status, social class were collected in the main interview.

##### Social

Social support was measured using the 4-item Medical Outcome Study Social Support Survey (MOS-SSS) [27]. Whether the participant was a lone adult in their household or not was collected in the main interview.

##### Violence perpetration

Violence perpetration was assessed using one question from the self-completion module used in the CSEW: “Have you ever used force or violence on anyone on purpose, for example, by scratching, hitting, kicking or throwing things, which you think injured them in some way? Please include your family and people you know, as well as strangers”.

##### Clinical

Diagnosis was used as a marker of illness severity, measured using the patient’s primary ICD-10 diagnosis (as recorded in their clinical notes or reported by their care co-ordinator); analysed as a binary variable of whether or not their diagnosis was of schizophrenia and related disorders (schizotypal and delusional). A history of any past admission under the MHA was also used as a marker of illness severity. Substance misuse was assessed by a question on whether the participant had ever taken drugs, and the World Health Organisation measure for hazardous alcohol use, the Alcohol Use Disorders Identification Test (AUDIT) [28].

### Ethics

Ethical approval for this study was obtained from National Research Ethics Service (NRES) Committee South East Coast-Kent in June 2011 (REC reference 11/LO/0672).

### Statistical analyses

Stata 13 (2013) was used for all analyses. All analyses were stratified by gender, as we were interested in comparing the strength and independence of associations in men and women. Logistic regression was used to explore the independence of associations between childhood maltreatment and adulthood domestic and sexual violence victimisation. We tested the association between any childhood maltreatment and each of the adulthood domestic and sexual violence victimisation using three logistic regression models:

1. Crude association.
2. Partially adjusted model (adjusting for age, ethnicity and social class).
3. Fully adjusted model (additionally adjusted for employment status, social support, living alone, perpetration of violence, diagnosis, admission under the Mental Health Act, alcohol and substance misuse).

We also tested confounder-adjusted associations between different types of childhood maltreatment and each of the adulthood domestic and sexual violence victimisation.

Descriptive statistics (proportions or means as appropriate) were used to describe the sample characteristics and proportion of missing data. As there was missing data on the exposure and outcome variables (see Table 1), reducing the analytic sample to individuals with complete data could have resulted in potentially biased estimates. To address this, we used multiple imputation. All of the variables of interest for the analyses listed in study variables were included in the imputation model, with 60 imputations used. All 318 participants with data on self-complete module were included in the imputation model. A sensitivity analysis was conducted to compare findings between the imputed dataset and the complete case analyses.

**Table 1:** Sample characteristics (*n* = 318)

Demographic	
Sex; *n* (%)	
Male	181 (56.92)
Female	137 (43.08)
Missing	0 (0)
Age	
Mean (SD)	41.76 (10.74)
Missing; *n* (%)	0 (0)
Ethnicity; *n* (%)	
White British	117 (36.79)
Not White British	200 (62.89)
Missing	1 (0.31)
Educational attainment; *n* (%)	
None	62 (19.50)
GCSEs	74 (23.27)
A-level/apprenticeship	59 (18.55)
Degree/diploma	85 (26.73)
Other	37 (11.64)
Missing	1 (0.31)
Employment status; *n* (%)	
Employed/student/inactive	66 (20.75)
Unemployed	252 (79.25)
Missing	0 (0)
Social class household reference person; *n* (%)	
High	44 (13.84)
Medium	69 (21.70)
Low	171 (53.77)
Missing	34 (10.69)
Social	
Lives alone; *n* (%)	
No	100 (31.45)
Yes	218 (68.55)
Missing	0 (0)
Perpetration of violence; *n* (%)	
No	199 (62.58)
Yes	95 (29.87)
Missing	24 (3.77)
Social support; *n* (%)	
Low	73 (22.96)
Medium	77 (24.21)
High	150 (47.17)
Missing	18 (5.66)
Clinical	
Diagnosis; *n* (%)	
Schizophrenia, schizotypal, and delusional	184 (57.86)
Bipolar affective disorder	40 (12.58)
Other mood disorders	30 (9.43)
Personality and behaviour disorders	26 (8.18)
Other disorders	30 (9.43)
Missing	8 (2.52)
Ever admitted under MHA; *n* (%)	
No	105 (33.02)
Yes	183 (57.55)
Missing	30 (9.43)
Ever taken drugs; *n* (%)	
No	93 (29.25)
Yes	204 (64.15)
Missing	21 (6.61)
AUDIT	
Non-hazardous drinking	171 (53.77)
Hazardous drinking	57 (17.92)
Missing	90 (28.30)
Victimisation	
Childhood maltreatment (any moderate to severe); *n* (%)	
No	82 (25.79)
Yes	146 (45.91)
Missing all 25 items	18 (5.66)
At least one item complete, less than 12	20 (6.30)
At least 12 items complete, less than 25	52 (16.35)
All 25 items answered	228 (71.70)
Adulthood domestic violence; *n* (%)	
No	112 (35.22)
Yes	149 (46.86)
Missing	57 (17.92)
Adulthood sexual violence; *n* (%)	
No	173 (54.40)
Yes	110 (34.59)
Missing	35 (11.01)

## Results

### Sample flow and characteristics

**Fig. 1:**
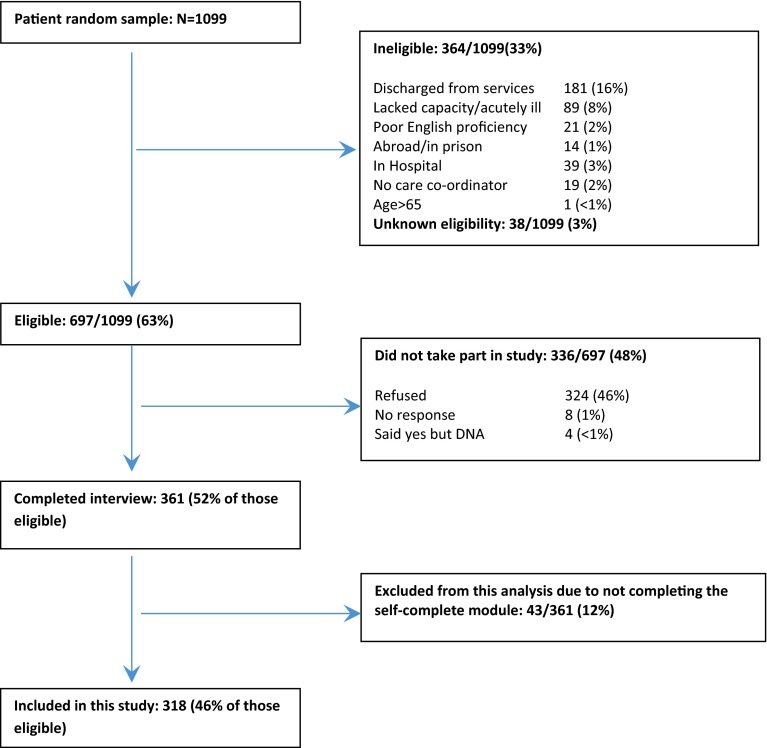
Sample flow

The sample flow of the original study is shown in Fig. 1. Three hundred and eighteen participants who had completed the self-completion module of the interview were included in the analyses.

The characteristics of the sample (including missing data for each variable) are presented in Table 1. There were slightly more men than women in the sample (57 %), and less than 40 % were White British. Fifty-eight percent of the sample had a diagnosis of schizophrenia or related disorder, and 58 % had a history of admission under the Mental Health Act.

### Prevalence of victimisation

**Fig. 2:**
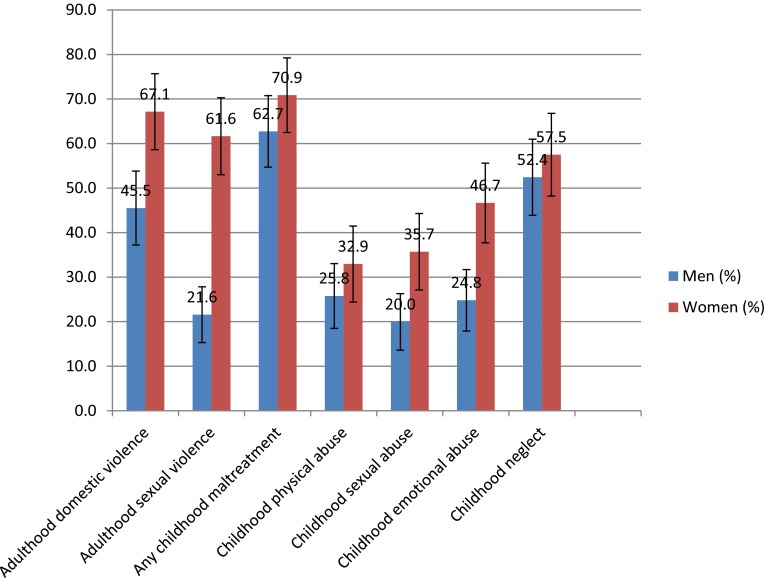
Prevalence and 95 % CIs of violent victimisation by gender (derived from imputed dataset, *n* = 318)

The prevalence of victimisation (both adulthood and childhood) for men and women is shown in Fig. 2. Childhood maltreatment was reported by 63 % (95 % CI 55–71 %) of men and 71 % (95 % CI 63–79 %) of women, 46 % (95 % CI 37–54 %) of men and 67 % (95 % CI 59–76 %) of women reported adulthood domestic violence victimisation, and 22 % (95 % CI 15–28 %) of men and 62 % (95 % CI 53–70 %) of women reported adulthood sexual violence victimisation.

### Association between childhood maltreatment and adulthood victimisation

The odds ratios and 95 % confidence intervals for adulthood domestic and sexual violence associated with experiencing moderate to severe childhood maltreatment, compared to no experience of childhood maltreatment, are shown in Table 2.

**Table 2:** Crude and adjusted odds for association of childhood maltreatment with adulthood domestic and sexual violence victimisation (*N* = 318)

Association with any moderate to severe childhood maltreatment	Unadjusted		Partially adjusted model^a^		Fully adjusted model^b^	
	OR (95 % CIs)	*p*	OR (95 % CIs)	*p*	OR (95 % CIs)	*p*
ADVV						
Men	4.32 (1.99–9.41)	<0.001	4.59 (2.06–10.22)	<0.001	3.75 (1.43–9.81)	0.007
Women	5.67 (2.22–14.54)	<0.001	6.05 (2.07–17.71)	0.001	6.29 (1.64–24.09)	0.007
ASVV						
Men	3.92 (1.42–10.83)	0.009	4.05 (1.43–11.49)	0.008	2.88 (0.89–9.34)	0.077
Women	2.22 (0.96–5.13)	0.061	2.43 (1.01–5.84)	0.048	2.28 (0.82–6.39)	0.116

^**a**^Adjusted for age, ethnicity and social class

^b^Adjusted for age, ethnicity, social class, employment status, social support, living alone, perpetration of violence, diagnosis, admission under Mental Health Act, alcohol and substance misuse

In the partially adjusted models, the odds of experiencing ADVV and ASVV were over four times higher among men who experienced childhood maltreatment than men without those experiences in childhood. For women who experienced childhood maltreatment, the odds of ADVV were increased over 6 times, and the odds of ASVV were over two times higher than women without experiences of childhood maltreatment. In the fully adjusted models, the strength of the association was not substantially altered in women, but was weaker in men.

### Different forms of childhood maltreatment

The odds ratios and 95 % confidence intervals associated with the effect of different forms of childhood maltreatment on ADVV and ASVV are shown in Table 3. For both men and women, each different form of childhood maltreatment was associated with increased odds for ADDV, with emotional abuse in childhood showing the strongest association with around eight times the odds in men and women. For both men and women, all forms of childhood abuse, but not childhood neglect, were significantly associated with increased odds of ASVV. For men, the strongest association with ASVV was sexual abuse in childhood, associated with around eight times the odds, however, for women this association was only three times, and emotional abuse had a slightly larger effect.

**Table 3:** Odds ratios of associations between different types of childhood abuse and neglect and adult domestic and sexual violence, stratified by gender

	Men		Women	
	Adjusted OR^a^ (95 % CIs)	*p*	Adjusted OR^a^ (95 % CIs)	*p*
ADVV				
Physical	3.76 (1.53–9.26)	0.004	3.84 (1.33–11.08)	0.013
Sexual	3.16 (1.19–8.39)	0.021	3.68 (1.32–10.23)	0.013
Emotional	8.44 (2.68–26.65)	<0.001	7.63 (2.56–22.79)	<0.001
Neglect	2.96 (1.37–6.42)	0.006	5.69 (2.11–15.31)	0.001
ASVV				
Physical	2.78 (1.17–6.62)	0.021	2.92 (1.18–7.26)	0.021
Sexual	7.72 (2.86–20.85)	<0.001	3.02 (1.22–7.51)	0.017
Emotional	5.22 (2.17–12.58)	<0.001	4.06 (1.72–9.58)	0.001
Neglect	2.20 (0.95–5.13)	0.066	2.06 (0.92–4.62)	0.077

NB all models are derived from an imputed dataset (*N* = 318)

^a^Adjusted for age, ethnicity and social class

### Sensitivity analyses

Sensitivity analyses were conducted using only those participants with complete data on these variables of interest, to compare results from the complete cases to those obtained with the imputed dataset. Complete case analyses are run for the main associations, as in Table 2, as well as the exploration of different forms of childhood maltreatment as in Table 3. We observed a similar pattern of associations in the complete case analysis as that obtained using the imputed dataset, but with wider confidence intervals reflecting the smaller sample size. Complete case analysis tables are available as supplementary material.

## Discussion

### Principal findings

We found a very high prevalence of childhood maltreatment and adulthood domestic and sexual violence victimisation among both men and women with SMI. For both men and women, experiences of moderate to severe childhood maltreatment were associated with two to six times the odds of domestic and sexual violence victimisation in adulthood. All forms of childhood abuse independently increased the odds of victimisation in adulthood. The strength of association between childhood maltreatment and adulthood domestic violence victimisation were similar for men and women. However, the association between childhood abuse and adulthood sexual violence victimisation appeared to be stronger in men than in women.

### Strengths and limitations

We used detailed, validated measures of childhood abuse and adulthood victimisation, and studied associations in both men and women. Most studies investigating domestic and sexual violence have exclusively focused on women and to date, the full picture of associations in both men and women has been unknown. We have shown that childhood abuse increases the odds of adult victimisation in both men and women with SMI. We were also able to investigate the effect of different forms of childhood maltreatment on adulthood violent victimisation, including emotional abuse and neglect, the effects of which have seldom been investigated.

Nevertheless, our study had limitations. The low response rate (52 %), may have introduced selection bias into the sample. Low response rates are a common challenge in studies of SMI participants, with ‘gatekeeping’ being a particular problem when participants are contacted through care co-ordinators, as was the case in this study. It is possible that people who have experienced victimisation would not want to take part in a study requiring disclosure of these experiences and this can lead to underestimation of the prevalence of victimisation. The prevalence of victimisation obtained in this study is in keeping with that reported in previous studies, which suggests that our sample was not particularly unusual.

Missing data on the variables of interest in this study is another limitation. With sensitive questions about abuse and victimisation, it is possible that data are not missing at random, as it is possible that people with a history of abuse may feel less comfortable answering questions about abuse than those without that experience. However, as there may be similar effects on answering questions referring to abusive experiences in childhood and adulthood, the effect of missingness may not bias the associations between childhood and adulthood abuse too greatly, and again the result would be to underestimate the strength of associations.

A further limitation is that all experiences of victimisation were self-reported and thus potentially subject to information bias. There is a possibility that those reporting adult victimisation may be more likely to recall and report childhood victimisation, as victimisation in adulthood may revive childhood memories, which would lead to an overestimation of the association. Generally, it has been found that people with SMI are reliable in their reports of childhood and adult abuse, although men with SMI may be less reliable regarding reports of sexual abuse [29]. It is thus possible that the lower prevalence of violence victimisation in men is due to under-reporting. A recent study using archival data of the minimization-denial subscale of the CTQ found that minimization of childhood maltreatment is common among community samples and psychiatric patients, and that this may underestimate associations between childhood maltreatment and associated outcomes [30].

As the study was a cross-sectional survey, it is not strictly possible to infer causality between the outcome and the exposure. Although reverse causality is unlikely, due to the time-bound nature of exposure (in childhood) and outcome (in adulthood), there may be many other explanations for the association observed, including recall bias and unmeasured confounding.

### Comparison with previous literature

The high prevalence of childhood abuse found in this study is similar to that previously reported in people with SMI [14, 15]. The prevalence of childhood neglect was very high in both women and men with SMI. This should be investigated in more detail in SMI populations, as neglect has been shown to have adverse mental health outcomes in the general population [31], and yet most studies with SMI populations have focused on abuse. The finding of an increased risk of domestic and sexual violence with experiences of childhood maltreatment is in line with previous research among populations with SMI [21, 22, 24]. Women with SMI with a history of childhood abuse were over three times more likely to experience adult sexual assault in one study [24]. Another study looking at men and women with SMI found that patients who had been sexually abused as adults were more likely to have sexually abused as children, but physical abuse in childhood was not associated with physical abuse in adulthood [22]. Other research has demonstrated the high prevalence of sexual and physical abuse among men and women with SMI, and the association with history of childhood abuse [21]. Our findings extend past literature by showing the association between childhood maltreatment and adulthood victimisation among men and women with SMI, and by exploring the effect of different forms of childhood maltreatment, such as emotional abuse and neglect, that previous studies have not investigated.

Many of the associations between childhood maltreatment and adulthood sexual violence victimisation were weaker among women than men. This is likely due to the difference in prevalence of sexual violence victimisation experiences in adulthood between men and women. As the majority of women in our sample (62 %) had experienced sexual violence, compared to 22 % of men, it is likely that specific childhood risk factors do not show such a strong association with what is sadly and shockingly such a prevalent outcome for women with SMI. Using an ecological framework to understand sexual and domestic violence victimisation [10], it follows that for women with SMI, there are so many risk factors for violence present, that the experience of childhood maltreatment may have less independent effect on vulnerability to later victimisation. Particularly for sexual violence, as it is less prevalent in men, it is likely that individual risk factors such as childhood maltreatment will have a greater effect on the risk of revictimisation.

The results of this study also suggest that emotional abuse in childhood increases the odds of adulthood domestic and sexual violence victimisation in both women and men. This is an important finding, as previous studies have neglected the effect of forms of childhood maltreatment other than physical and sexual abuse on adulthood victimisation. This finding adds to the evidence suggesting that rather than there being something about specific (i.e. physical and sexual) abusive experiences in childhood that makes people more vulnerable to similar abusive experiences in adulthood, broader stressful childhood experiences may affect the life-trajectory negatively in terms of complex social and behavioural outcomes which may increase vulnerability to violence. Indeed, one large prospective study in the general population found that childhood maltreatment had little direct impact on lifetime mental health outcomes when stressful life events were controlled for [31].

### Implications

As abusive experiences in both childhood and adulthood are associated with poorer mental health outcomes in people with SMI, the findings of this study highlight the importance of clinicians rigorously assessing patients’ trauma history, and addressing the complex needs of patients who have been victimised. The risk of re-traumatisation in institutional settings as well as private must be considered. Interventions to address victimisation in adulthood do not take early experiences into account. Future research is therefore needed to understand the mechanism linking childhood maltreatment and adulthood victimisation. It may be the case that experiences of victimisation in early life influence risk of later victimisation in this causal manner, via changes in social and psychological development and the severity of illness. On the other hand, the association may simply reflect continuity of adversity across the life course, with early victimisation a marker of social disadvantage that is still present in adulthood and increasing risk of victimisation. Interventions aimed at helping people with SMI deal with the consequences of traumatic experiences may lead to improved clinical outcomes. NICE guidelines state that trauma-focused psychological treatment should be offered to PTSD sufferers regardless of the time that has elapsed since the trauma [32]. People with SMI who have been victims of childhood maltreatment should potentially be considered candidates for such treatment. The results of this study also highlight the importance of addressing a wider range of traumatic experiences in childhood, such as emotional abuse, which have previously been neglected.

## References

[1] Maniglio R (2009). Severe mental illness and criminal victimization: a systematic review. Acta Psychiatr Scand.

[2] Khalifeh H, Johnson S, Howard LM, Borschmann R, Osborn D, Dean K (2015). Violent and non-violent crime against adults with severe mental illness. Br J Psychiatry.

[3] Coid J, Petruckevitch A, Feder G, Chung W, Richardson J, Moorey S (2001). Relation between childhood sexual and physical abuse and risk of revictimisation in women: a cross-sectional survey. Lancet.

[4] Widom CS, Czaja SJ, Dutton MA (2008). Childhood victimization and lifetime revictimization. Child Abuse Negl.

[5] Miller BJ, Paschall CB, Svendsen DP (2006). Mortality and medical comorbidity among patients with serious mental illness. Psychiatr Serv.

[6] Osborn DP, Levy G, Nazareth I, Petersen I, Islam A, King MB (2007). Relative risk of cardiovascular and cancer mortality in people with severe mental illness from the United Kingdom’s General Practice Rsearch Database. Arch Gen Psychiatry.

[7] Khalifeh H, Dean K (2010). Gender and violence against people with severe mental illness. Int Rev Psychiatry.

[8] Khalifeh H, Moran P, Borschmann R, Dean K, Hart C, Hogg J (2015). Domestic and sexual violence against patients with severe mental illness. Psychol Med.

[9] Patrick K (2013). It’s time to put maternal suicide under the microscope. CMAJ.

[10] Grauerholz L (2000). An ecological approach to understanding sexual revictimization: linking personal, interpersonal, and sociocultural factors and processes. Child Maltreat.

[11] Field NP, Classen C, Butler LD, Koopman C, Zarcone J, Spiegel D (2001). Revictimization and information processing in women survivors of childhood sexual abuse. J Anxiety Disord.

[12] Classen CC, Palesh OG, Aggarwal R (2005). Sexual revictimization: a review of the empirical literature. Trauma Violence Abuse.

[13] Kendall-Tackett KA, Williams LM, Finkelhor D (1993). Impact of sexual abuse on children: a review and synthesis of recent empirical studies. Psychol Bull.

[14] Mueser KT, Goodman LB, Trumbetta SL, Rosenberg SD, Osher FC, Vidaver R (1998). Trauma and posttraumatic stress disorder in severe mental illness. J Consult Clin Psychol.

[15] Rosenberg SD, Lu W, Mueser KT, Jankowski MK, Cournos F (2007). Correlates of adverse childhood events among adults with schizophrenia spectrum disorders. Psychiatr Serv.

[16] Muenzenmaier K, Schneeberger AR, Castille DM, Battaglia J, Seixas AA, Link B (2014). Stressful childhood experiences and clinical outcomes in people with serious mental illness: a gender comparison in a clinical psychiatric sample. J Fam Violence.

[17] Bak-Klimek A, Karatzias T, Elliott L, Campbell J, Pugh R, Laybourn P (2014). Nature of child sexual abuse and psychopathology in adult survivors: results from a clinical sample in Scotland. J Psychiatr Ment Health Nurs.

[18] Teplin LA, McClelland GM, Abram KM, Weiner DA (2005). Crime victimization in adults with severe mental illness: comparison with the National Crime Victimization Survey. Arch Gen Psychiatry.

[19] Goodman LA, Rosenberg SD, Mueser KT, Drake RE (1997). Physical and sexual assault history in women with serious mental illness: prevalence, correlates, treatment, and future research directions. Schizophr Bull.

[20] Oram S, Trevillion K, Feder G, Howard LM (2013). Prevalence of experiences of domestic violence among psychiatric patients: systematic review. Br J Psychiatry.

[21] Goodman LA, Salyers MP, Mueser KT, Rosenberg SD, Swartz M, Essock SM (2001). Recent victimization in women and men with severe mental illness: prevalence and correlates. J Trauma Stress.

[22] Coverdale JH, Turbott SH (2000). Sexual and physical abuse of chronically ill psychiatric outpatients compared with a matched sample of medical outpatients. J Nerv Ment Dis.

[23] Bengtsson-Tops A, Markstrom U, Lewin B (2005). The prevalence of abuse in Swedish female psychiatric users, the perpetrators and places where abuse occurred. Nord J Psychiatry.

[24] Cloitre M, Tardiff K, Marzuk PM, Leon AC, Portera L (1996). Childhood abuse and subsequent sexual assault among female inpatients. J Trauma Stress.

[25] Charlwood P MA, Goldacre M, Cleary R, Wilkinson E (eds) (1999) Health outcome indicators: severe mental illness. Report of a working group to the Department of Health. Oxford: National Centre for Health Outcomes Development

[26] Bernstein DP, Stein JA, Newcomb MD, Walker E, Pogge D, Ahluvalia T (2003). Development and validation of a brief screening version of the Childhood Trauma Questionnaire. Child Abuse Negl.

[27] Sherbourne CD, Stewart AL (1991). The MOS social support survey. Soc Sci Med.

[28] Saunders JB, Aasland OG, Babor TF, de la Fuente JR, Grant M (1993). Development of the alcohol use disorders identification test (AUDIT): who collaborative project on early detection of persons with harmful alcohol consumption–II. Addiction.

[29] Goodman LA, Thompson KM, Weinfurt K, Corl S, Acker P, Mueser KT (1999). Reliability of reports of violent victimization and posttraumatic stress disorder among men and women with serious mental illness. J Trauma Stress.

[30] MacDonald K, Thomas ML, Sciolla AF, Schneider B, Pappas K, Bleijenberg G (2016). Minimization of childhood maltreatment is common and consequential: results from a large, multinational sample using the childhood trauma questionnaire. PLoS One.

[31] Horwitz AV, Widom CS, McLaughlin J, White HR (2001). The impact of childhood abuse and neglect on adult mental health: a prospective study. J Health Soc Behav.

[32] National Institute for Health and Care Excellence (NICE) (2005) Nice guidelines [CG26] Post-traumatic stress disorder: management 31211536

